# The effect of vitamin D deficiency on platelet parameters in patients with COVID-19

**DOI:** 10.3389/fcimb.2024.1360075

**Published:** 2024-03-08

**Authors:** Amirhossein Talebzadeh, Hadi Ghaffari, Kazem Ghaffari, Sorur Yazdanpanah, Bahman Yousefi Goltappeh, Majid Eslami, Ali Ghasemi

**Affiliations:** ^1^Department of Biochemistry and Hematology, Semnan University of Medical Sciences, Semnan, Iran; ^2^Department of Bacteriology and Virology, Semnan University of Medical Sciences, Semnan, Iran; ^3^Department of Base and Laboratory Sciences, Khomein University of Medical Sciences, Khomein, Iran; ^4^Department of Hematology and Blood Banking, School of Allied Medical Sciences, Shahid Beheshti University of Medical Sciences, Tehran, Iran; ^5^Department of Immunology, Semnan University of Medical Sciences, Semnan, Iran; ^6^Cancer Research Center, Semnan University of Medical Sciences, Semnan, Iran

**Keywords:** platelets, COVID-19, vitamin D, thrombocytosis, mean platelet volume

## Abstract

**Introduction:**

Since there is very little information about the relationship between platelet parameters and vitamin D concentration in patients with COVID-19, the aim of this study is to investigate the relationship between serum vitamin D level and platelet parameters in patients with COVID-19 and to compare these parameters in patients with COVID-19 without vitamin D deficiency and, subsequently, the prognostic value of these parameters in cases of vitamin D deficiency.

**Methods:**

Seven hundred and forty-three patients diagnosed with COVID-19 were enrolled in this study. Patients were divided into two groups: those with and without vitamin D deficiency. The associations between platelet indices and vitamin D levels were analyzed by Pearson’s correlation analysis and a one-way ANOVA test.

**Results:**

Platelet count and mean platelet volume (MPV) were significantly higher in the patients with vitamin D deficiency than in the patients without vitamin D deficiency. There was a significant negative correlation between platelet count and MPV with vitamin D levels in patients with vitamin D deficiency (r = -0.835, P = 0.001 & r = -0.324, P = 0.042, respectively). Vitamin D levels in COVID-19 patients can determine the platelet count and MPV of the patients.

**Discussion:**

The aforementioned results imply that maintaining an elevated concentration of vitamin D in COVID-19 patients is important because it is associated with a decrease in MPV, which in turn reduces susceptibility to diseases such as coronary artery disease.

## 1 Introduction

In December 2019, a new coronavirus named “SARS-CoV2” was declared by the World Health Organization as the cause of the outbreak of COVID-19 (Li et al., 2021). In fact, COVID-19 is the third epidemic disease with respiratory manifestations after Sars and MERS syndromes (Li et al., 2021), which, along with higher contagiousness (Yu et al., 2020), was able to infect most countries of the world, including Iran (Zhang et al., 2020).

Vitamin D deficiency has been widely reported in different countries as a factor that may cause excessive inflammation and dysfunction of the immune system and may also have adverse effects on hemostasis and thrombosis (Bodnar et al., 2014). Recently, the vitamin D receptor has been found in PLTs, which plays an essential role in anti-thrombogenicity. In addition, vitamin D deficiency has been found to be associated with changes in PLT size (Korzonek-Szlacheta et al., 2018). Vitamin D deficiency is considered a serious public health problem that affects all populations of all ages in developed and developing countries. There is very little published information about the relationship between PLT function and changes in its indices with vitamin D concentration in patients with COVID-19. However, in previous studies, the relationship between high MPV and low vitamin D levels has been shown in patients with stable coronary artery disease, patients without chronic disease, and pregnant women with gestational diabetes mellitus (Cure et al., 2014; Gur et al., 2015; Park et al., 2017; Korzonek-Szlacheta et al., 2018). In some studies, it has been suggested that PLT parameters such as MPV can be used as a prognostic marker in sepsis, severe diseases and also in the disease of COVID-19 (Liu et al., 2020; Zhong and Peng, 2021).

Prognostic importance as well as changes in platelet (PLT) indices have been shown in some diseases such as metabolic syndrome, cardiovascular attacks, ischemic attacks, myocardial infarction, and hepatitis C infection (Pohl et al., 2001; Coban et al., 2006; Wojszel et al., 2008; Ateş et al., 2009; Meng et al., 2016).

Since there is very little information about the relationship between PLT parameters and vitamin D concentration in patients with COVID-19, the aim of this study is to investigate the relationship between serum vitamin D level and PLT parameters in patients with COVID-19 and to compare these parameters in patients with COVID-19 without vitamin D deficiency and, subsequently, the prognostic value of these parameters in cases of vitamin D deficiency. Increased understanding of PLT functions in COVID-19 has undoubtedly improved our knowledge about clinical management and treatment options and may lead to the development of more accurate treatment strategies.

## 2 Materials and methods

### 2.1 Study subjects and biochemical measurements

Seven hundred and forty-three patients with diagnosed COVID-19 who were referred to the Khordad Hospital, Varamin, Iran, were included in this matched case-control study. Finally, patients with diagnosed COVID-19 were divided into two groups with and/or without vitamin D deficiency. Vitamin D levels were dichotomized between normal vitamin D levels and vitamin D deficiency according to the standard cut point of 20 ng/mL (Meltzer et al., 2020). A control group of outpatients from the same hospital was selected in the same time period to compare the results with the patient group.

Cases and controls were frequency matched on gender, body mass index (BMI), systolic/diastolic blood pressure, total cholesterol, fasting glucose, triglycerides, white blood cell, hemoglobin, smoking status, race, and C-reactive protein.

The result of the COVID-19 test in the control group was negative. All patients who had a complete initial blood count and biochemical tests were included in the study.

PLT count, PLT distribution width (PDW), MPV, plateletcrit (PCT), and lymphocyte count were automatically determined by using an automated blood cell counter (Mindray, BC-6800, Mindray Biomedical Electronics, Nanchang, Shenzhen, China). Aspartate aminotransferase (AST) was measured by spectrophotometric technique via the Pars Azmun kit (Karaj, Iran). The serum level of 25-OH-vitamin D (ng/mL) was measured with the enzyme‐linked immunosorbent assay method (EUROIM-MUN^®^, D-23560 Lubeck, Germany). AST-to-PLT ratio index (APRI = [AST/upper limit of normal] × [100/PLTs, ×109/L]), vitamin D*MPV, vitamin D*PLT, vitamin D*PDW, PLT-lymphocyte ratio (PLR), MPV/PLT count ratio (MPR), MPV to lymphocyte ratio (MLR), MPV/PCT, PDW/PCT, PDW/PLT and BMI; [weight/height squared (kg/m2)] were calculated. The variables of the control group were matched with the control group.

### 2.2 Inclusion and exclusion criteria

The inclusion criteria for the study included patients with COVID-19 with a definitive diagnosis by molecular method (PCR) without any history of disease leading to vitamin D deficiency. Exclusion criteria: (1) patients who had a primary sclerosing cholangitis, primary biliary cirrhosis, Wilson’s disease, hemochromatosis, pancreatitis, pancreatic cancer, bile duct cancer, ovarian cancer, endometrial cancer, breast cancer, lung cancer, esophageal and stomach cancer, patients with simultaneous infection of two or more types of hepatitis, a history of heart disease that leads to the use of antiplatelet drugs, pregnant women with a history of preeclampsia, and chronic and incurable diseases that affect the PLT count or PLT indices; (2) patients with uncontrolled diabetic mellitus; (3) comorbidity and (4) use of antiplatelet drugs such as aspirin and non-steroidal anti-inflammatory drugs.

### 2.3 Statistical analysis

Statistical analyses were performed using SPSS 25.0 software (Inc., Chicago, IL, USA) and a genetic analyzer (ABI PRISM 310, Applied Biosystems). The mean and standard deviation (mean ± SD), Pearsonʼs χ^2^ test and one-way ANOVA test were used to compare the two groups’ characteristics. Additionally, in order to evaluate the association between PLT parameters and serum vitamin D levels, the parameters that demonstrated a P-value less than 0.1 in the bivariable correlation analysis were incorporated into the binary logistic regression analysis model. Pearson’s correlation coefficient was used for assessing the relationships between PLT parameters and vitamin D levels. P < 0.05 was considered a statistical difference.

## 3 Results

Biochemical tests and the complete blood count of 36 patients were incomplete; thus, these subjects were excluded from the study. A total of 707 patients were included in the study. Patients were divided into two groups: 345 COVID-19-infected patients with vitamin D deficiency and 362 COVID-19-infected patients without vitamin D deficiency. The mean ± SD age of the patients was 54.8 ± 13.2 years. Three hundred and forty-one patients (48.2%) were male, and 366 patients (51.8%) were female. The gender distribution was similar in the studied groups. About 91% of all studied patients were vaccinated.

The mean ± SD vitamin D level of the patients was 24.1 ± 9.3 ng/mL, ranging from 11.2–52.1 ng/mL. The mean level of vitamin D in females and males was 33.4 ± 10.6 ng/mL and 38.8 ± 11.3 ng/mL, respectively, which showed a significant difference (P < 0.001), so that the mean level of vitamin D in females was lower than that of males. In total, thrombocytopenia occurred in 11.8% of patients with COVID-19. The demographic characteristics of three study groups are summarized in **Table 1**.

**Table 1:** Demographic and clinical characteristics of patient.

Characteristics	Patients with vitamin D deficiency (N = 345)	Patients without vitamin D deficiency (N = 362)	Control subjects (100)	P_*_	P_**_	P_***_
Gender, n (%) Male Female	166 (48.1) 179 (51.2)	175 (48.3) 187 (51.6)	48 (48) 52 (52)	.952	.948	.952
Age, years ± SD	61.8 ± 10.5	48.6 ± 12.3	45.1 ± 11.7	.031	.004	.597
Mean weight ± SD (kg)	77.4 ± 30.3	75.2 ± 28.6	78.2 ± 23.1	.724	.631	.573
Mean body mass index ± SD (kg/m2)	27.5 ± 3.8	26.4 ± 3.1	26.6 ± 2.9	.719	.639	.827
ECOG^c^ performance status 0-1 2	296 (85.8) 49 (14.2)	323 (89.2) 39 (10.8)		.167		
Fever max, (°C)	39.8 ± 0.6	39.5 ± 0.4		.211		
Thorax computed tomography, Positive, n (%)	273 (79.1)	302 (83.4)		.143		
VitD (ng/mL)	17.1 ± 8.2	34.4 ± 10.3	35.2 ± 12.6	.004	<.001	.598

Data are No. (%) unless otherwise indicated. SD; Standard of deviation, n; number. ^c^ECOG; Eastern Cooperative Oncology Group.

P* Patients with vitamin D deficiency vs. patients without vitamin D deficiency.

P** Patients with vitamin D deficiency vs. control subjects.

P*** Patients without vitamin D deficiency vs. control subjects.

There was no clinically significant difference between the study groups in gender, weight, body mass index, Eastern Cooperative Oncology Group, fever, or thorax computed tomography (P > 0.05); however, there was a significant difference in serum vitamin D levels between the groups. In the control subjects, serum vitamin D levels were significantly higher compared to the COVID-19-infected patients (**Table 1**).

The values of the studied variables in all three groups are shown in **Table 2**. Based on laboratory findings regarding PDW and AST, there were no significant differences between groups. The MPR, MLR, PCT, PDW/PCT, and lymphocytes showed no difference between the groups of COVID-19-infected patients with and without vitamin D deficiency (P > 0.05).

**Table 2:** Comparison of platelet parameters in three groups.

Laboratory values	Patients with vitamin D deficiency (N = 345)	Patients without vitamin D deficiency(N = 362)	Control subjects (100)	P_*_	P_**_	P_***_
Mean PLT ± SD (×10^3^/μl) Min-Max	318.2 ± 48.4 110.2-481.3	219.4 ± 56.6 100.5-375.2	266.1 ± 45.9 101.7-396.4	.021	.042	.033
MPV ± SD, fL	10.0 ± 0.6	8.6 ± 0.5	8.5 ± 0.9	.038	.038	.967
MPR	.031	.039	.032	.133	.229	.727
Mean lymphocyte ± SD (×10^3^/μl)	1.12 ± 8.5	.94 ± 9.2	2.82 ± 7.7	.369	<.001	<.001
MLR	8.9	9.1	3.0	.107	<.001	<.001
PLR	284.1 ± 101.5	233.4 ± 132.3	94.3 ± 129.4	.029	<.001	<.001
PCT, %	0.20	0.20	0.20	.999	.999	999
MPV/PCT	50.0	43.0	38.6	.148	.034	.129
PDW, %	16.7	16.2	16.2	.234	.109	.927
PDW/PCT	83.6	81.0	73.6	.241	.027	.047
PDW/PLT	.04	.07	.06	.046	.103	.965
Vitamin D*MPV	171.0	295.8	299.2	.002	.002	.341
Vitamin D*PLT	5441.2	7547.3	9366.7	.011	<.001	.023
Vitamin D*PDW	285.5	557.2	570.2	<.001	<.001	.982
Mean AST ± SD, (IU/L)	30.1 ± 10.2	31.3 ± 12.3	27.8 ± 9.7	.667	.387	.255
APRI	.09	.14	.10	.037	.738	.072

PLT, platelet count; PCT, Plateletcrit; MPV, mean platelet volume; MPR, MPV/platelet count ratio; MLR, MPV to lymphocyte ratio; PLR, platelet-lymphocyte ratio; AST, aspartate aminotransferase; PDW, platelet distribution width; APRI, AST-to-PLT ratio index. * Denote the multiplication operation and the product is the average concentration of serum vitamin D in each group in each of the desired variables.

P* Patients with vitamin D deficiency vs. Patients without vitamin D deficiency.

P** Patients with vitamin D deficiency vs. Control subjects.

P*** Patients without vitamin D deficiency vs. Control subjects.

**Figure 1:**
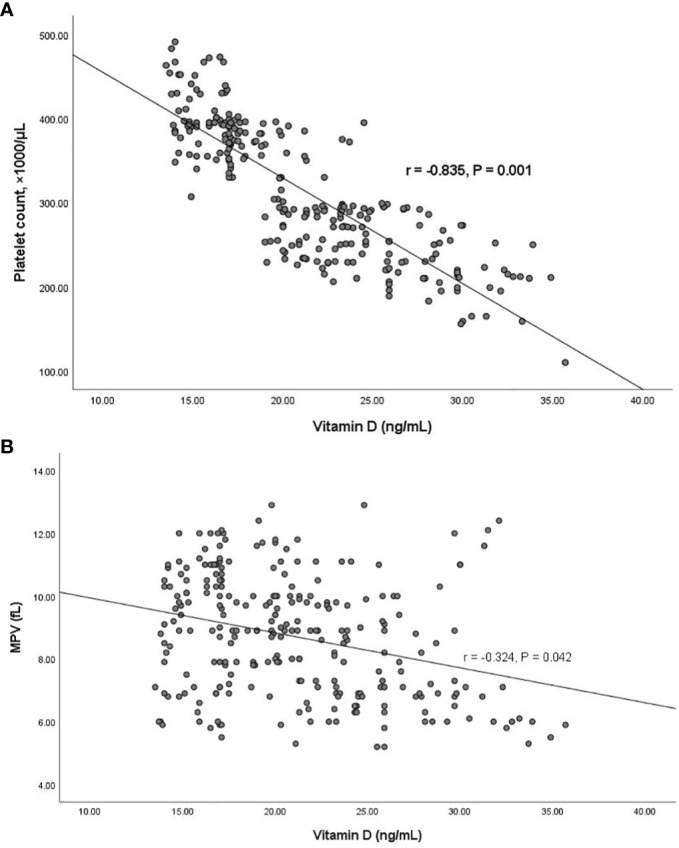
**(A)** The negative correlation between platelet count and vitamin D levels. **(B)** The negative correlation between MPV and vitamin D levels. P-value was calculated by Pearson’s correlation.

Using Pearson’s correlation coefficient test, we investigated the correlation between PLT count and MPV with vitamin D levels in the group of patients with vitamin D deficiency. As shown in **Figures 1A, B**, there was a significant negative correlation between PLT count and MPV with vitamin D levels in patients with vitamin D deficiency (r = -0.835, P = 0.001, & r = -0.324, P = 0.042, respectively).

**Figure 2:**
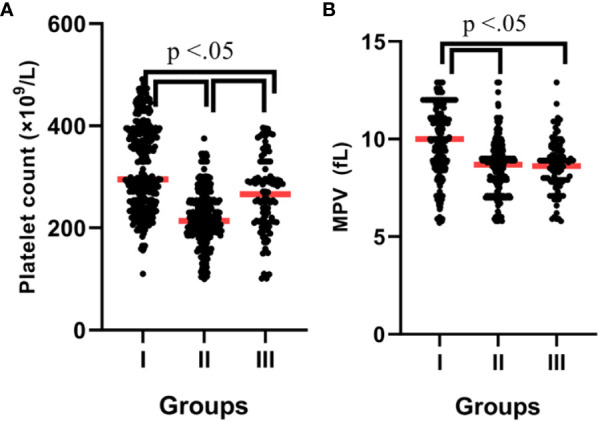
**(A)** Platelet count according to groups. **(B)** MPV according to groups. P-values were calculated by ANOVA. I; Patients with vitamin D deficiency, II; Patients without vitamin D deficiency, III; Control subjects.

The PLT count displayed significant differences between the groups, being greatest in the COVID-19-infected patients with vitamin D deficiency and least in the COVID-19-infected patients without vitamin D deficiency; COVID-19-infected patients with vitamin D deficiency vs. control subjects [378.2 ± 48.4 vs. 266.1 ± 45.9, P = 0.042], and COVID-19-infected patients with vitamin D deficiency *vs.* COVID-19-infected patients without vitamin D deficiency [378.2± 48.4 *vs.* 219.4± 56.6, P = 0.021] (**Figure 2A**).

As shown in **Figure 2B**, the MPV was significantly higher in the patients with vitamin D deficiency than in the patients without vitamin D deficiency (10.0 ± 0.6 *vs.* 8.6 ± 0.5, P = 0.038). Also, the MPR was significantly higher in the patients without vitamin D deficiency than in the patients with vitamin D deficiency (0.39 *vs.* 0.26, P = 0.023). The PDW/PCT and MLR were significantly lower in the control subjects than in the patients (P > 0.05). In addition, several complex parameters caused by serum vitamin D levels with MPV, PLT, and PDW are shown in **Table 2**.

Two sets of multiple linear regressions were performed, considering PLT parameters as the dependent variables and serum vitamin D levels and age as the independent variables. The first linear regression model (**Table 3**), using PLT count and vitamin D and age, exhibited that there is a relationship between PLT count with age and vitamin D serum level. But no relationship was found between PLT count and gender. Also, the first linear regression model, using MPV as a dependent variable and vitamin D as a regressor, showed that there is a relationship between MPV and vitamin D serum levels. A second adjusted linear regression model was performed, confirming the existence of an independent association between PLT count and MPV and serum vitamin D levels, even after adjusting for age (**Table 3**).

**Table 3:** Multivariate analysis for the association with PLT counts and MPV.

Variable	Model 1; PLT counts		Model 2; PLT counts	
	Odds ratio (95% CI)	*P*	Odds ratio (95% CI)	*P*
Age (yr)	1.12 (1.03-1.31)	0.095	1.19 (1.07-1.39)	0.071
Vitamin D (ng/ml)	0.93 (0.79-0.96)	0.021	0.97 (0.83-0.99)	0.011
	Model 1; MPV		Model 2; MPV	
Age (yr)	0.45 (0.14-1.40)	0.135	0.39 (0.17-1.51)	0.201
Vitamin D (ng/ml)	1.26 (0.61-1.45)	0.035	1.45 (1.17-4.01)	0.041

PLT, platelet count; MPV, mean platelet volume, OR; Odds ratios and 95% CI indicates; confidence interval.

## 4 Discussion

In this study, it was observed that the PLT count in COVID-19-infected patients with vitamin D deficiency was significantly higher than the other two groups. In other words, vitamin D deficiency increased the PLT count. Also, we showed in this study that there was a significant negative correlation between PLT count and MPV with vitamin D levels in COVID-19-infected patients.

Consistent with our results, in a study on Korean adults, the authors reported that the subjects with vitamin D deficiency had a significantly lower PLT and MPV than the sufficiency group (Park et al., 2017). In another study, Alanli et al. showed that PLT counts increased in people with low vitamin D levels (Alanli et al., 2020).

Vitamin D deficiency is a worldwide problem, with billions of people suffering from vitamin D deficiency (Amrein et al., 2020). Also, the prevalence of vitamin D deficiency in Iranian men and women is very high, especially in old age (Vatandost et al., 2018).

The correlation results between MPV and vitamin D levels have been inconsistent in different studies. Some studies have documented an inverse relationship between MPV and levels of vitamin D in different diseases, while others have failed to observe any association between these two variables. For example, a study on gestational diabetes mellitus found that low 25-hydroxyvitamin D3 levels and high MPV were observed in pregnant women with gestational diabetes mellitus (Gur et al., 2015). On the other hand, Cumhur et al (Chu et al., 2022). & Alanli et al. (2020) reported that there was no significant relationship between vitamin D deficiency and MPV levels in healthy participants.

Aihara et al. (2004) provided evidence indicating that the vitamin D receptor (VDR) system may have a significant role in the prevention of blood clotting *in vivo*. They demonstrated this by observing that mice lacking the VDR gene (VDRKO mice) experienced an intensified formation of blood clots in multiple organs after being injected with lipopolysaccharide, regardless of their levels of calcium. On the other hand, the activation of vitamin D increased the gene expression of antithrombotic factors and thrombomodulin in monocytic cells while decreasing the expression of the thrombogenic factor gene. In the VDRKO mice, the opposite effect was observed. Therefore, the vitamin D-VDR system promotes the expression of antithrombotic factors and inhibits the expression of thrombogenic factors.

Megakaryocytes, which serve as precursors for PLTs, possess VDRs as well. The activation of these receptors plays a role in governing cell maturation and the proliferation of megakaryocytes. In the context of vitamin D deficiency, the promotion of megakaryocyte maturation and the elevation of PLT counts can be observed (Silvagno et al., 2010).

Furthermore, low levels of vitamin D have been associated with increased levels of proinflammatory cytokines such as interleukin-6 (IL-6) and tumor necrosis factor alpha (TNF-α), which in turn may lead to increased MPV (Cure et al., 2014). Increased levels of IL-6 and TNF-α can stimulate megakaryopoiesis and platelet production, and they may have effects on hematopoietic stem cells (Kaser et al., 2001; Chu et al., 2022). On the other hand, studies have shown that IL-6 and TNF-α levels increase in COVID-19 patients (Coomes and Haghbayan, 2020).

Hence, it is reasonable to propose that one of the reasons for the increase in MPV and PLT counts may potentially be attributed to the severe manifestation of the cytokine storm in COVID-19 patients who show vitamin D deficiency.

In some studies, it has been suggested that PLT parameters can be used as a prognostic marker in sepsis, critical illnesses, and also in COVID-19 (Liu et al., 2020; Zhong and Peng, 2021), but despite this, contradictory results are seen in different studies. By evaluating different studies, it was found that two factors can be mentioned among the reasons for observing these contradictions: one is the difference in measurement techniques, and the other is the clinical conditions of the patients. Among the differences in measurement techniques, we can mention the anticoagulants used to collect the sample and the duration of sample storage until the test. All these factors may affect PLT parameters, including MPV levels (Harrison and Goodall, 2016). Among the differences in the clinical conditions of the patients, we can mention the deficiency or decrease in the level of vitamin D in the patients, gender, and HPA polymorphisms (Gur et al., 2015; Alanli et al., 2020; Ghaffari et al., 2023).

There have been no studies that clearly show an increase in PC in COVID-19 patients with vitamin D deficiency. In this study, it was shown that although the mean PLT count in COVID-19 patients without vitamin D deficiency did not show thrombocytopenia, the mean PLT count in these patients was significantly lower than that of normal individuals, which can be due to various reasons, including abnormal hematopoiesis and the initiation of an auto-immune response against PLTs in COVID-19 patients (Jolicoeur and Lamontagne, 1995; Yang et al., 2005).

Our study had several limitations. The evidence suggests that calcium & magnesium deficiency may contribute to low vitamin D status (Dai et al., 2018; Khazai et al., 2008). Therefore, calcium & magnesium deficiency can indirectly cause an increase in MPV and PLT counts. Hence, it is suggested to measure calcium & magnesium levels as well. Also, we did not measure the level of pro-inflammatory cytokines in this study, while measuring their level can help us understand the mechanisms involved in the increase of MPV and PLT counts.

An increased number of platelets has been associated with an elevated likelihood of thrombosis in COVID-19 patients. In a study of 3915 hospitalized COVID-19 patients, it was found that patients with an elevated platelet count (>400 × 10^9/L) had an increased risk of critical illness and all-cause mortality (Barrett et al., 2021).

This study is simply a report of retrospective clinical data. However, it has nothing new to offer about mechanisms and interrelations. The interpretation of platelet role in COVID-19 is complicated. Platelets are key regulator of immune system in viral infections. COVID-19 patients platelets show higher activity. However complex pathophysiology of COVID-19 and cytokine storm in infection background makes it harder to study immune cells (including platelets) role in COVID-19 infection. It becomes more complicated when we think about relationship of vitamin D with platelets.

## 5 Conclusion

Vitamin D levels in COVID-19 patients can determine the platelet count and MPV of the patients. Vitamin D deficiency increased the number of PLT. Also, there is a significant negative correlation between the number of PLT and MPV with the level of vitamin D in patients with Covid-19.

## Data availability statement

The raw data supporting the conclusions of this article will be made available by the authors, without undue reservation.

## Ethics statement

Ethical principles were followed based on the ethical protocol approved by the Ethics Committee at Semnan University of Medical Sciences, Semnan, Iran (IR.SEMUMS.REC.1401.309). The studies were conducted in accordance with the local legislation and institutional requirements. The participants provided their written informed consent to participate in this study.

## Author contributions

AT: Data curation, Investigation, Writing – original draft. HG: Conceptualization, Methodology, Visualization, Writing – original draft. KG: Software, Validation, Writing – original draft. SY: Formal analysis, Methodology, Software, Writing – original draft. BY: Project administration, Resources, Supervision, Writing – original draft. ME: Funding acquisition, Validation, Writing – original draft. AG: Conceptualization, Methodology, Supervision, Validation, Writing – original draft, Writing – review & editing.
